# Effects of community-based interventions for stillbirths in sub-Saharan Africa: a systematic review and meta-analysis

**DOI:** 10.1016/j.eclinm.2023.102386

**Published:** 2023-12-22

**Authors:** Uchenna Gwacham-Anisiobi, Yebeen Ysabelle Boo, Adetola Oladimeji, Jennifer J. Kurinczuk, Nia Roberts, Charles Opondo, Manisha Nair

**Affiliations:** a National Perinatal Epidemiology Unit, Nuffield Department of Population Health, University of Oxford, Oxford, United Kingdom; b Solina Center for International Development and Research, Nigeria; c Bodleian Health Care Libraries, University of Oxford, Oxford, United Kingdom; d Department of Medical Statistics, London School of Hygiene and Tropical Medicine, London, United Kingdom

**Keywords:** Community-based intervention, Fetal death, Stillbirth, Effectiveness, Sub-Saharan Africa

## Abstract

**Background:**

Sub-Saharan Africa (SSA) alone contributed to 42% of global stillbirths in 2019, and the rate of stillbirth reduction has remained slow. There has been an increased uptake of community-based interventions to combat stillbirth in the region, but the effects of these interventions have been poorly assessed. Our objectives were to examine the effect of community-based interventions on stillbirth in SSA.

**Methods:**

In this systematic review and meta-analysis, we searched eight databases (MEDLINE [OvidSP], Embase [OvidSP], Cochrane Central Register of Controlled Trials, Global Health, Science Citation Index and Social Science Citation index [Web of Science Core Collection], CINAHL [EBSCOhost] and Global Index Medicus) and four grey literature sources from January 1, 2000 to July 7, 2023 for relevant studies from SSA. Community-based interventions targeting stillbirths solely or as part of complex interventions, with or without hospital interventions were included, while hospital-only interventions, microcredit schemes and maternity waiting home interventions were excluded. Study quality was assessed using the Cochrane risk of bias and National Heart, Lung and Blood Institute's tools. The study outcome was odds of stillbirth in intervention versus control communities. Pooled odds ratios (ORs) were estimated using random-effects models, and subgroup analyses were performed by intervention type and strategies. Publication bias was evaluated by funnel plot and Egger’s test. This study is registered with PROSPERO, CRD42021296623.

**Findings:**

Of the 4223 records identified, seventeen studies from fifteen SSA countries were eligible for inclusion. One study had four arms (community only, hospital only, community and hospital, and control arms), so information was extracted from each arm. Analysis of 13 of the 17 studies which had community-only intervention showed that the odds of stillbirth did not vary significantly between community-based intervention and control groups (OR 0.96; 95% CI 0.78–1.17, I^2^ = 57%, p ≤ 0.01, n = 63,884). However, analysis of four (out of five) studies that included both community and health facility components found that in comparison with community only interventions, this combination strategy significantly reduced the odds of stillbirth by 17% (OR 0.83; 95% CI 0.79–0.87, I^2^ = 11%, p = 0.37, n = 244,868), after excluding a study with high risk of bias. The quality of the 17 studies were graded as poor (n = 2), fair (n = 9) and good (n = 6).

**Interpretation:**

Community-based interventions alone, without strengthening the quality and capacity of health facilities, are unlikely to have a substantial effect on reducing stillbirths in SSA.

**Funding:**

Nuffield Department of Population Health, 10.13039/501100006558Balliol College, the 10.13039/501100014748Clarendon Fund, 10.13039/501100000265Medical Research Council.

**Research in context**

**Evidence before this study**

A systematic review and meta-analysis of the effect of community-based interventions was conducted by including published and unpublished literature between January 1, 2020 and July 7, 2023 using the following search terms: “community-based interventions”, “stillbirths”, “perinatal death”, “sub-Saharan Africa”, “pregnancy”, “birth attendants”, “home visits”, “women’s groups”, “mobile health”, “health promotion”, “nutritional interventions”, “smoking cessation”. Several studies conducted globally have tested the effect of different types of interventions on stillbirths. The Lassi and Bhutta 2015 Cochrane review of global community-based interventions which included 26 randomised controlled trials reported a 19% reduction in the risk of stillbirths, however, in this review, some included studies had components of hospital-based intervention. There is no published systematic review or meta-analysis evaluating the effectiveness of community-based interventions on stillbirths focusing on sub-Saharan Africa alone.

**Added value of this study**

The study highlights the importance of integrated efforts in reducing stillbirths. Community-based interventions alone have limited impact, emphasising the need for coordination, collaboration, and shared responsibility across different healthcare levels, including both community and hospital settings.

**Implications of all the available evidence**

The study’s findings can serve as a basis for formulating evidence-based policies and guidelines for stillbirth prevention in sub-Saharan Africa. The study shows that community-based interventions alone have limited impact in reducing stillbirths in the region. Therefore, it is crucial to combine these initiatives targeting pregnant women and their families with increased investment in healthcare infrastructure, human resources, and services. This integration will help accelerate the reduction of stillbirths.

## Introduction

According to the World Health Organisation (WHO), a baby who dies at 28 weeks of gestation or later, but before or during birth is classified as a stillbirth. Nearly two million babies were stillborn in 2019,^1^ and 98% of the deaths occurred in low-middle income countries (LMICs).^1^ Sub-Saharan Africa (SSA) alone contributed to 42% of global stillbirths in 2019, with Nigeria accounting for the third highest number globally.^2^ Although the cause of stillbirths in many empirical studies are unknown largely due to inadequate documentation/capturing of women’s health conditions and circumstances before stillbirth,^3^ some biomedical conditions have been associated with increased risk. These include antepartum haemorrhage, maternal conditions leading to placental insufficiency (e.g., diabetes, hypertensive disorders), infections (e.g., Malaria, HIV, Syphilis and Group B Streptococcus), complications during labour and childbirth (e.g., obstructed labour, uterine rupture, umbilical cord complications, etc) and genetic disorders.^4^, ^5^, ^6^

Several interventions which have the potential for preventing stillbirths by intervening along the causal pathway of the causes of stillbirths have been implemented in various parts of SSA. Bhutta and colleagues in 2009 classified global interventions for stillbirth prevention into four, depending on the timing of its implementation along the maternal health continuum: interventions before pregnancy, during pregnancy, in labour, and during childbirth.^7^ These interventions involve a range of services targeting women and health workers and can be implemented in hospitals or communities. Although the regional and global policy recommendations for SSA is for women to have skilled antenatal care provision, and skilled support during childbirth, preferably in a health facility; only 22.3% of women in SSA give birth in health facilities,^8^ and 76% have a skilled antenatal check-up.^9^ Given the sustained burden of stillbirths in SSA and the enormous barriers to quality care access such as cost, poor care experience, distance and delays in skilled intervention during emergencies,^10^, ^11^, ^12^, ^13^ it is not surprising that several community-based interventions have been implemented either as stand-alone projects or as part of health system-wide interventions in many communities in SSA.^14^^,^^15^ We defined community-based interventions as multicomponent interventions that generally combine individual, behavioural and environmental change strategies extending from households into participant’s localities aiming to prevent dysfunction, promote well-being among women and enhance maternal and newborn outcomes.^16^ Common types of community interventions in SSA include nutritional interventions, infection prevention and treatment, improving access to skilled childbirth and behavioural interventions.^17^

However, despite decades of implementing community-based interventions for maternal and newborn health (including stillbirths), little progress has been made to date. The annual reduction rate for stillbirth in SSA was 1.4% between 2000 and 2019.^18^ Furthermore, transferable insights from effective interventions across communities and countries in this region were often lost due to poor reporting of interventions and inadequate assessment of intervention acceptability.^17^

This article reports part of a wider systematic review undertaken by the authors to identify the types, reporting, effectiveness and acceptability of community-based prevention of stillbirths in SSA. The types, reporting and acceptability of the interventions were presented in a separate paper^17^; this paper presents a narrative synthesis and meta-analysis of the effectiveness of community-based intervention, implemented alone or in conjunction with a hospital-based component, on the incidence of stillbirth in SSA.

## Methods

### Search strategy and selection criteria

A systematic review and meta-analysis following our study protocol published in PROSPERO (CRD42021296623) was conducted and reported using the PRISMA guideline. In brief, following the search of eight databases and four grey literature sources for relevant studies published between 1 January 2000 and 7 July 2023, and the retrieved reports were assessed for eligibility using the study inclusion and exclusion criteria. The databases included were: MEDLINE (OvidSP) [1946–present], Embase (OvidSP) [1974–present], Cochrane Central Register of Controlled Trials (Cochrane Library, Wiley) [1ssue 7 of 12, July 2022], Global Health (OvidSP) [1973–2022 week 28], Science Citation Index and Social Science Citation index (Web of Science Core Collection) [1900–present], CINAHL (EBSCOhost) [1982–present] and Global Index Medicus https://www.globalindexmedicus.net/) and four grey literature sources (ProQuest Dissertations and Theses–Global, www.who.int/trialsearch/, www.ClinicalTrials.gov and Google (conference proceedings and implementation reports).

Details of the inclusion and exclusion criteria used for the complete review is described in a separate paper.^17^ Briefly, reports which described community-based interventions targeting stillbirth as a sole or part of a complex intervention, with or without a health facility-based component were included in the review project. However, in this part of the review, only the studies which assessed the effectiveness of the intervention/s on the incidence of stillbirths were included. Hospital only interventions and maternal waiting home interventions were excluded as women in both cases had daily access to health workers^19^ and were not in their communities. Also, socioeconomic interventions which could have an indirect impact on stillbirths such as micro credit women empowerment schemes were excluded, however, studies which provided vouchers for women to ease transportation to health facilities were included.

Development of the search strings was supported by a librarian (NR). The search terms used were synonyms and specific terms from different types of related interventions implemented in the community in similar reviews found during the scoping search. They included “community-based interventions”, “stillbirths”, “perinatal death”, “sub-Saharan Africa”, “pregnancy”, “birth attendants”, “home visits”, “women’s groups”, “mobile health”, “health promotion”, “nutritional interventions”, “smoking cessation”. UGA developed the initial search strategy, which was reviewed by MN and finalised by the University Librarian NR. The search terms and search strings were customised to each database, register and search engine. Boolean operators “OR” between synonyms and “AND” between search strings were applied to widen the scope of the search without losing its focus.^20^ The search terms were piloted on MEDLINE, the inclusion and exclusion criteria were applied to five percent of the papers retrieved to check the reliability for identifying relevant papers. The full search strategy is shown in the Supplementary Material (Appendix 1) and is also described in a previous paper.^17^ The search was first conducted on December 16, 2021 by UGA and reviewed by NR. An updated search was conducted on July 7, 2023.

Three reviewers UGA, YYB and AO independently screened the records retrieved from the database and grey literature search to identify the papers included in the review. Disagreements in the decision to include or exclude an article were resolved by consensus and unresolved disagreements were resolved by discussions with MN, CO, JJK. Included articles were assessed for quality using the Cochrane risk of bias and the National Heart, Lung and Blood Institute's tools. The overall quality rating for cluster randomised controlled trials (cRCTs) and randomised controlled trials (RCTs) was based on the assessment rating of all five domains of the risk of bias tool. For the pre-post design studies, a score of 1 was given to each question on the tool where the characteristics assessed were present ad zero was given if the characteristic was absent. Papers scoring 70% and above of possible scores were rated as good quality, while papers that scored 50%–69% and 0%–49% were rated as fair and poor quality, respectively (Appendix 2). Similar scoring and rating techniques were utilised for cross-sectional and cohort studies included in this review.

### Data analysis

Data extraction was independently conducted by UGA, YYB, and AO. Information about the study design, intervention type, number of participants, number of stillbirths and effect size were extracted to a pre-tested tool designed in Microsoft Excel (Appendix 3). None of the authors of included papers were contacted for additional information, as the review team believed that they had sufficient information to conduct the analysis. The main outcome was odds of stillbirths in the intervention versus control communities. The interventions were grouped by intervention types and intervention delivery strategies as discussed in the first part of this comprehensive review.^17^ The studies were categorised into solely community-based interventions and combined community-and-hospital interventions. For each group, a meta-analysis was conducted using random-effects model to estimate the pooled odds ratio (OR) of stillbirth in intervention communities compared with the control with 95% confidence intervals (95% CIs). Subgroup analyses were performed to estimate pooled OR by intervention type and intervention delivery strategy. The meta-analyses were done with all studies included, and a sensitivity analysis was conducted by excluding any poor-quality studies. The I^2^ test statistic and 95% CI and effect estimates were utilised to assess heterogeneity of effect estimates across studies. Publication bias was evaluated using a funnel plot and Egger’s test. All analyses were conducted using Stata SE, v17.

### Role of the funding source

The funders of the study had no role in study design, data collection, data analysis, data interpretation, or writing of the report. UGA, YYB, AO, NR, JJK, CO and MN had access to the data in the study and accept responsibility for the decision to submit for publication.

## Results

**Fig. 1:**
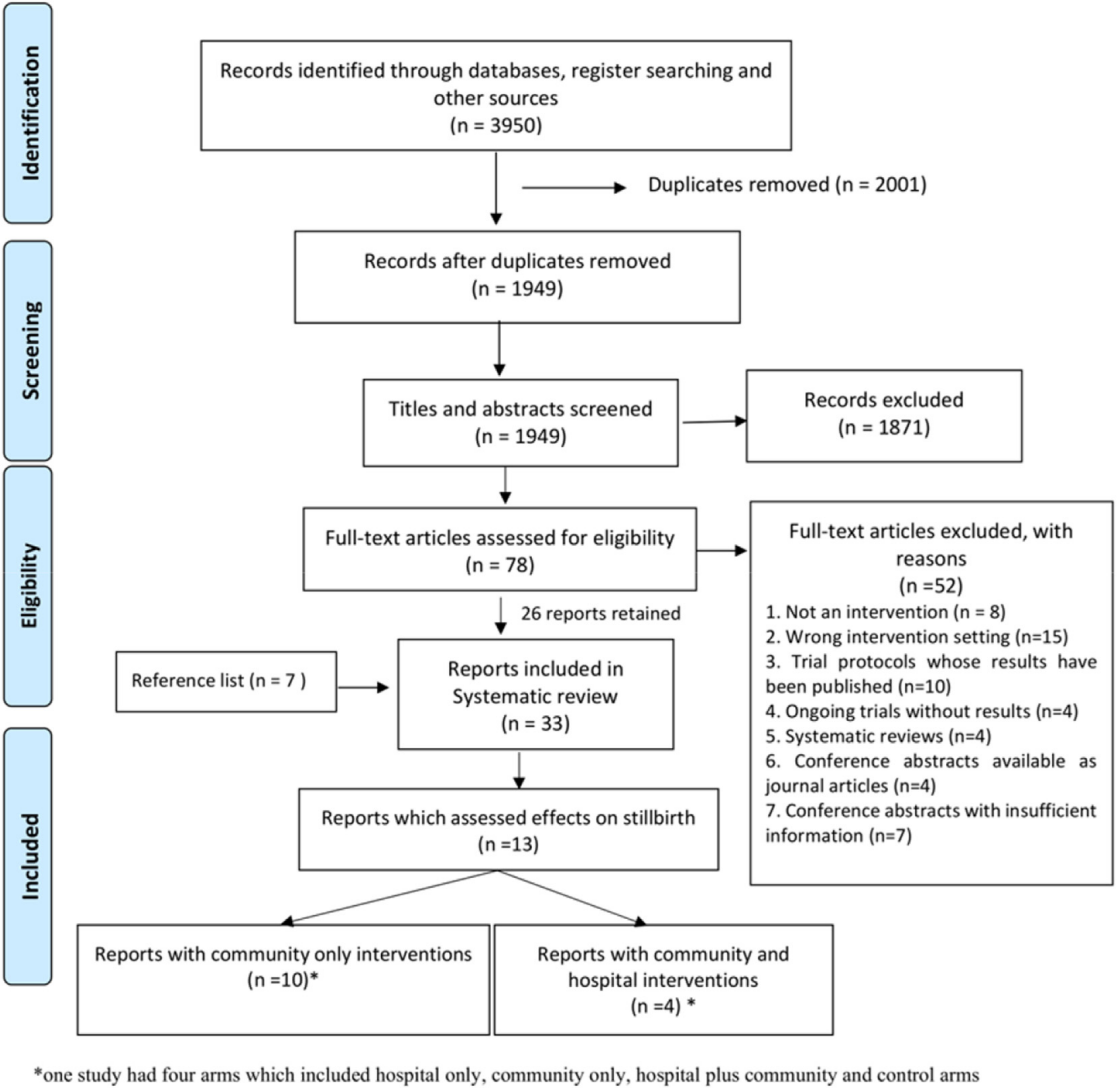
The PRISMA chart showing the systematic search and inclusion of studies.

The searches yielded a total of 4223 records (Fig. 1). Following deduplication, the title and abstracts of 2098 records were screened independently by three reviewers and 77 were retained for full-text review. After full text review, 45 records were excluded based on the inclusion/exclusion criteria and 7 further records were included after reference list searches. Of the 39 papers included in the systematic review, the 17 papers which quantitatively reported effectiveness of community-based interventions on incidence of stillbirths were included in this study (Table 1). Some retrieved reports assessed the effects of intervention on the composite indicator perinatal deaths (stillbirths and early neonatal deaths which is the death of neonates in the first seven days of life) but were excluded from this analysis to avoid selection bias as the effect estimates for stillbirths could not be separated from the total effect on perinatal deaths. Stillbirth was defined in all studies following the WHO definition for international comparison—babies born with no signs of life at 28 weeks or more of gestation.^38^

**Table 1:** Summary characteristics of all included studies.

	Title	First author and year	Country	Study design	Place of intervention
1	A Randomized Cluster Trial to Evaluate the Effect of Iodized Salt Exposure on Birth Outcome and Infant Development in Ethiopia	Mohammed, 2016^21^	Ethiopia	cRCT	Community only
2	Effects of quality improvement in health facilities and community mobilization through women's groups on maternal, neonatal and perinatal mortality in three districts of Malawi: MaiKhanda, a cluster randomized controlled effectiveness trial	Colbourn, 2013^22^	Malawi	cRCT	Community and hospitals
3	Reducing maternal and neonatal deaths in rural Malawi: evaluating the impact of a community-based women's group intervention	Lewycka, 2011^23^	Malawi	cRCT	Community only
4	Mobile Phone Intervention Reduces Perinatal Mortality in Zanzibar: Secondary Outcomes of a Cluster Randomized Controlled Trial	Lund, 2014^24^	Tanzania	cRCT	Community only
5	Reduced perinatal mortality following enhanced training of birth attendants in the Democratic Republic of Congo: a time-dependent effect	Matendo, 2011^25^	DRC	cRCT	Community only
6	Community-based malaria screening and treatment for pregnant women receiving standard intermittent preventive treatment with sulfadoxine-pyrimethamine: A multicenter (The Gambia, Burkina Faso, and Benin) cluster-randomized controlled trial	Scott, 2019^26^	Gambia, Burkina Faso and Benin	cRCT	Community only
7	Effect of personalized home-based support for pregnant women on pregnancy outcomes: a cluster randomized trial	Ilboudo, 2022^27^	Burkina Faso	cRCT	Community and hospitals
8	Improving coverage of antenatal iron and folic acid supplementation and malaria prophylaxis through targeted information and home deliveries in Côte d’Ivoire: a cluster randomised controlled trial	Kone, 2023^28^	Côte d’Ivoire	cRCT	Community only
9	Pregnancy outcomes and ethanol cook stove intervention: A randomized-controlled trial in Ibadan, Nigeria	Alexander, 2018^29^	Nigeria	RCT	Community only
10	Prenatal fortified balanced energy-protein supplementation and birth outcomes in rural Burkina Faso: A randomized controlled efficacy trial	De Kok, 2022^30^	Burkina Faso	RCT	Community only
11	Impact of traditional birth attendant training in Mozambique: a controlled study	Gloyd, 2001^31^	Mozambique	Non randomised CT	Community only
12	Impact of the Saving Mothers, Giving Life approach on decreasing maternal and perinatal deaths in Uganda and Zambia	Serbanescu, 2019^32^	Uganda and Zambia	Pre post	Community and hospitals
13	Advancing Survival in Nigeria: A Pre-post Evaluation of an Integrated Maternal and Neonatal Health Program	Sloan, 2018^33^	Nigeria	Pre-post	Community and hospitals
14	The effect of the community midwifery model on maternal and newborn health service utilization and outcomes in Busia County of Kenya: a quasi-experimental study	Shikuku, 2020^34^	Kenya	Pre post	Community only
15	Evaluating the impact of maternal health care policy on stillbirth and perinatal mortality in Ghana; a mixed method approach using two rounds of Ghana demographic and health survey data sets and qualitative design technique	Azaare, 2022^35^	Ghana	Pre-post (mixed)	Community only
16	Effectiveness of community-based support for pregnant women living with HIV: A cohort study in South Africa	Fatti, 2016^36^	South Africa	Cohort	Community only
17	Impact of a maternal and newborn health results-based financing intervention (RBF4MNH) on stillbirth: a cross-sectional comparison in four districts in Malawi	Makuluni, 2021^37^	Malawi	Cross sectional	Community and hospitals

cRCT: cluster randomised controlled trial; nrCT: non-randomised controlled trial; RCT: randomised controlled trial.

The 17 reports included were from studies conducted in 15 countries in SSA (Table 1). Three studies each were from Malawi^23^^,^^37^^,^^39^ and Burkina Faso,^26^^,^^27^^,^^30^ and the rest were from 13 other countries. Two studies were conducted in two or more countries.^26^^,^^32^ There was at least one study from all four regions of SSA. Seven studies were from west Africa conducted in Nigeria,^29^^,^^33^ Burkina Faso,^26^^,^^27^^,^^30^ Ghana,^35^ and Côte d’Ivoire.^28^ Equally, east Africa had seven studies, from Malawi,^23^^,^^37^^,^^39^ Tanzania,^24^ Ethiopia,^21^ Mozambique,^31^ Uganda and Zambia.^32^ There was one study each from central (Democratic Republic of Congo^25^) and southern Africa (South Africa^36^).

Eight of the included studies were cluster Randomised Controlled Trials (cRCT),^21^^,^^23^, ^24^, ^25^, ^26^, ^27^, ^28^^,^^39^ four had pre-post design,^32^, ^33^, ^34^, ^35^ two were RCT,^29^^,^^30^ while one each were non-randomised controlled trial (non-RCT),^31^ cohort^36^ and cross-sectional^37^ [Table 1]. Of the 17 studies, twelve were conducted solely in the community, four were conducted both in the communities and health facilities while one study had four arms comprising community only, community and health facility, health facility only and control. The interventions in the health facilities included one or more of the following: training health workers, purchasing equipment and employing adjunct medical staff.

### Effect of community-based interventions on stillbirths

Thirteen community only studies assessed the effect of the interventions on stillbirths. Of these, seven were RCTs, and one each were non-RCT, cohort and pre-post studies [Table 2]. Five studies were rated as having good quality; seven had fair quality, and one was rated as poor quality.

**Table 2:** Characteristics of community only intervention studies included in the meta-analysis.

	First author and year	Study design	Key intervention(s)	Control	Type of intervention	Intervention strategy	Quality rating
1	Mohammed, 2016	cRCT	Early access to Iodized salt distribution	Later access to Iodized salt	Nutritional	Community mobilisation	Fair
2	Colbourn, 2013	cRCT	Participatory women groups to improve care practices and health-seeking behaviours using quality improvement methods	Usual care	Knowledge and behavioural Increasing access to skilled childbirth	Women groups/peer counselling Volunteer and community Health worker training Community mobilisation	Good
3	Lewycka, 2011	cRCT	Women groups, peer health counselling, home visits	Usual care	Knowledge and behavioural	Women groups/peer counselling	Fair
4	Lund, 2014	cRCT	Automated and directed health messaging. Call voucher system for women to reach health workers.	Usual care	Knowledge and behavioural Increasing access to skilled birth	mHealth	Fair
5	Matendo, 2011	cRCT	Emergency newborn care training for TBAs, nurses and midwives.	Usual care	Increasing access to skilled childbirth	Traditional birth attendant training (TBA) Community mobilisation	Fair
6	Scott, 2019	cRCT	Malaria screening and treatment, home visits.	Usual care	Prevention and management of infections	Home visits Volunteer and community Health worker training	Good
7	Kone, 2023	cRCT	Home visits, nutritional counselling, micronutrient supplementation.	Usual care	Nutritional, Knowledge and behavioural	Home visits Volunteer and community Health worker training	Fair
8	De Kok, 2022	RCT	Provision of prenatal fortified balanced energy-protein supplement, iron and folic acid supplementation	Usual care	Nutritional, Knowledge and behavioural	Home visits Volunteer and community Health worker training	Good
9	Alexander, 2018	RCT	Provision of clean cook ethanol stoves, home visits and health education.	Usual care	Knowledge and behavioural	Home visits	Good
10	Gloyd, 2001	nRCT	Periodic TBA re/training and provision of essential supplies.	One off national TBA training	Increasing access to skilled childbirth	Traditional birth attendant training	Fair
11	Fatti, 2016	Cohort	Home visits, health education, counselling for anti-retroviral therapy initiation and adherence.	N/A	Prevention and management of infections	Home visits	Fair
12	Shikuku, 2020	Pre post	Community midwifery, training and deployment of health workers to hard to reach areas.	N/A	Increasing access to skilled childbirth	Community midwifery Volunteer and community Health worker training Community mobilisation	Poor
13	Azaare, 2022^28^	Pre-post (mixed)	National health policy for free maternal health care	Women accessing care prior to policy	Increasing access to skilled childbirth	Community mobilisation	Good

cRCT: cluster randomised controlled trial; nrCT: non-randomised controlled trial; RCT: randomised controlled trial; N/A: not applicable; TBA: traditional birth attendant.

**Fig. 2:**
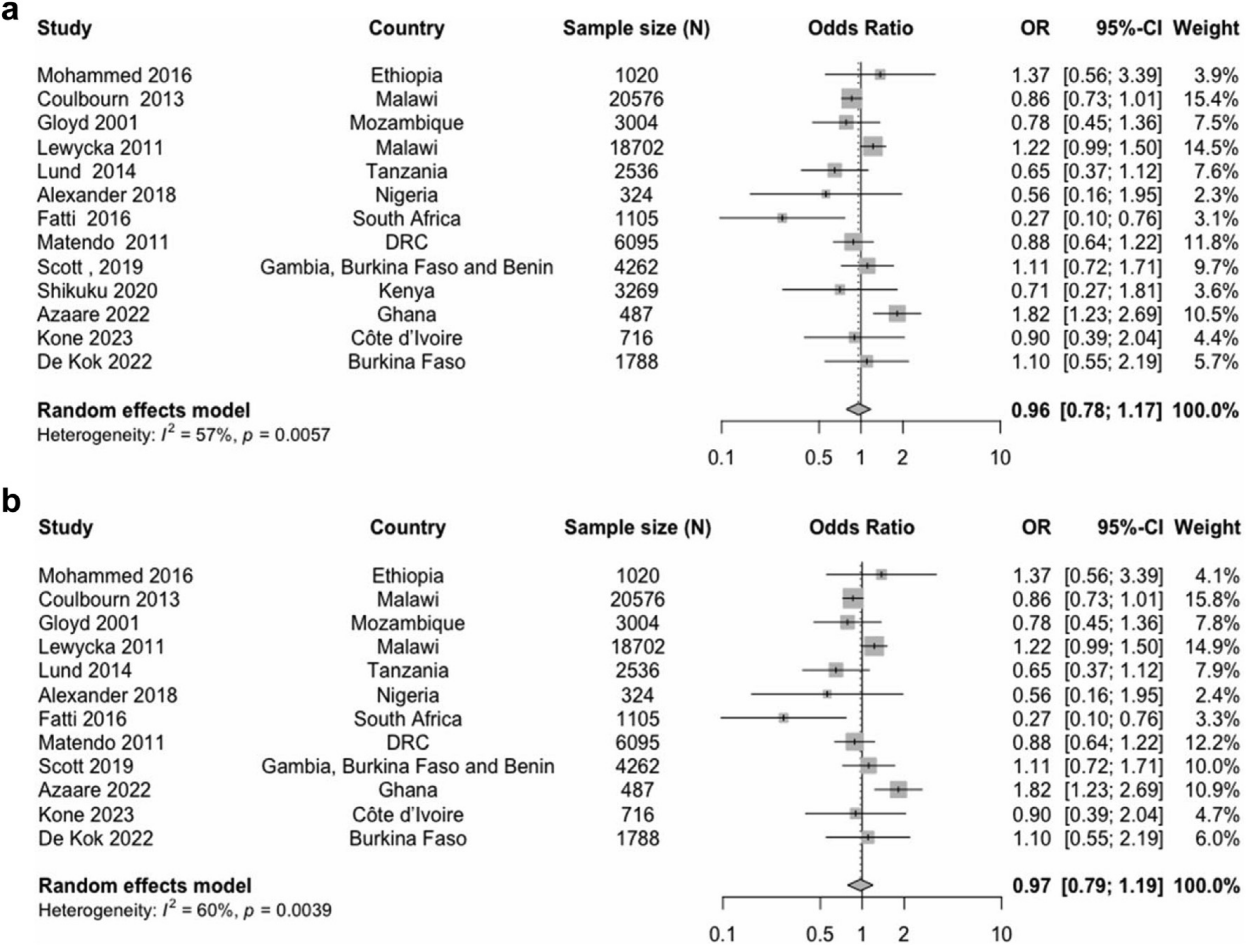
Forest plot of odds of stillbirths in intervention communities compared with control communities in (a) all studies (b) without high risk of bias study.

The meta-analysis including all thirteen studies of community-based intervention alone showed that the intervention communities had only marginally reduced odds of stillbirth compared with the control communities (OR 0.96; 95% CI 0.78–1.17, n = 63,884), which was not statistically significant (Fig. 2). Statistical heterogeneity across studies was moderate (I^2^ = 57%, p = 0.0057). A sensitivity analysis excluding the study with high risk of bias^34^ did not materially change the results (OR 0.97; 95% CI 0.79–1.19, n = 60,615).

**Fig. 3:**
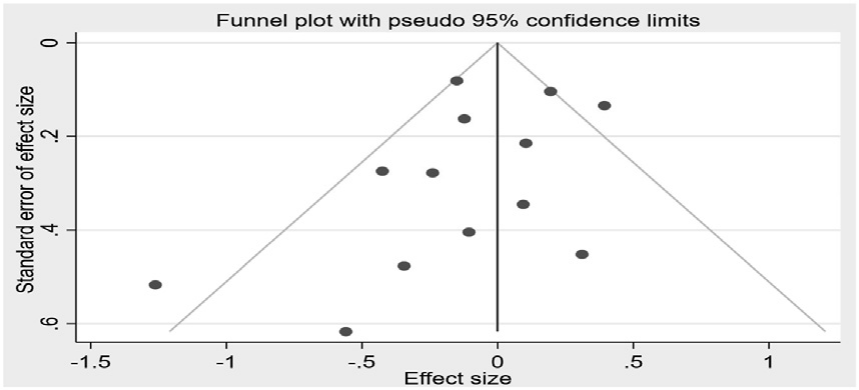
Publication bias evaluated by funnel plot.

As evidenced by the Funnel plot (Fig. 3) and Egger’s test (p = 0.08), there was a very weak evidence of publication bias.

### Subgroup analysis

As described in our previous paper,^17^ the researchers grouped interventions into four types based on the overall aim of the intervention. These were nutritional; infection screening, prevention and treatment; improving women’s health knowledge and behaviour; and improving access to skilled childbirth.^17^ Additionally, based on how the researchers went about achieving their research aims, the studies were categorised into nine intervention delivery strategies. These were women’s groups, training traditional birth attendants, provision of transportation vouchers, mobile phone based interventions, volunteer and community health worker training, community mobilisation, and mass media sensitisation.^17^ The details are included in a separate paper^17^ and are summarised in Panel 1.

Panel 1 **Summary of included studies by type of intervention and delivery strategy.**

**Table:** 

**Type of intervention**	
1	Nutritional
2	Prevention and management of infections (including HIV or Malaria)
3	Knowledge and behavioural (Educating for ANC4, Pollution, danger signs recognition)
4	Increasing access to skilled birth
**Intervention delivery strategy**	
1	mHealth
2	Women groups/peer counselling
3	community Midwifery
4	Home visits (including education, health screening and infection treatment)
5	Mass media
6	Traditional Birth Attendant (TBA) training
7	Volunteer and community Health worker training
8	Community mobilisation (including advocacy, health sensitization, transport and emergency loan scheme)
9	Transport voucher/transfers

**Fig. 4:**
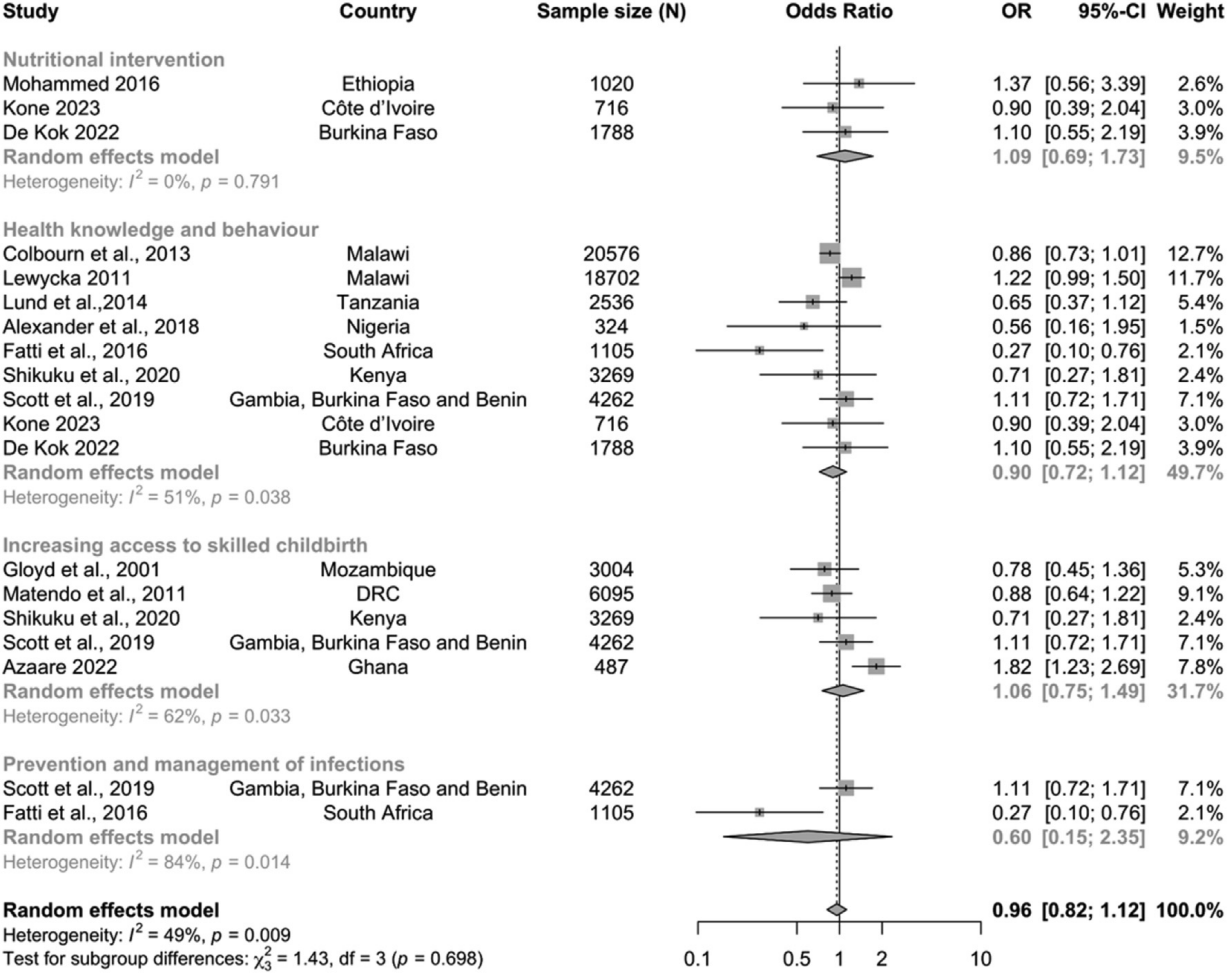
Forest plot of odds of stillbirths in intervention communities compared with control communities by intervention types.

The included studies implemented one or more types of intervention, using two or more intervention strategies. For example, the Colbourn study^22^ implemented two types of intervention (knowledge/behavioural intervention and increasing access to skilled childbirth) using three delivery strategies (women groups, volunteer/community health worker training and community mobilisation). To calculate the pooled effect by intervention types, the effect of studies which had more than one type of intervention were included in the pooled subgroup analysis for each intervention type (Fig. 4). The meta-analysis showed that compared with the control group, there was no evidence of lower odds of stillbirth for infection prevention and treatment (OR 0.60; 95% CI 0.15–2.35, n = 5367), health knowledge and behaviour (OR 0.90; 95% CI 0.72–1.12, n = 53,278), skilled attendance at childbirth (OR 1.06; 95% CI 0.75–1.49, n = 17,117), and nutritional interventions (OR 1.09; 95% CI 0.69–1.73, n = 3524). The heterogeneity in the subgroups varied from 0% (p = 0.79) to 84% (p = 0.014).

**Fig. 5:**
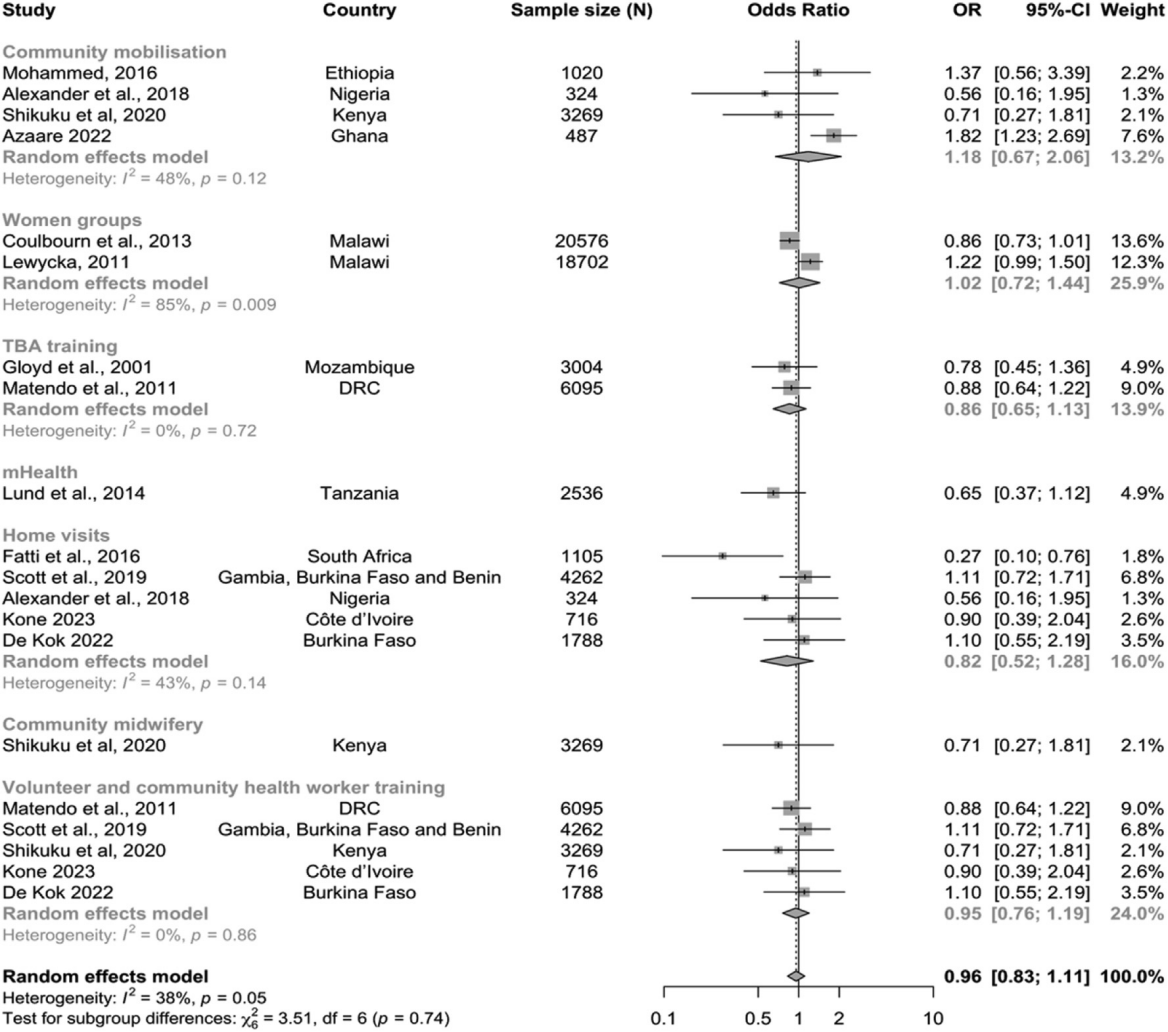
Forest plot of odds of stillbirths in intervention communities compared with control communities by intervention delivery strategies.

Subgroup analysis based on the intervention delivery strategy followed the same pattern as the previous analysis (Fig. 5). The meta-analysis showed that in all but two intervention delivery strategies (women groups and community mobilisation), the intervention communities also had non-significant lower odds of stillbirth, while women group ad community mobilisation interventions had no change. The heterogeneity in the subgroups varied from 0% (p = 0.79) to 84% (p = 0.014).

**Fig. 6:**
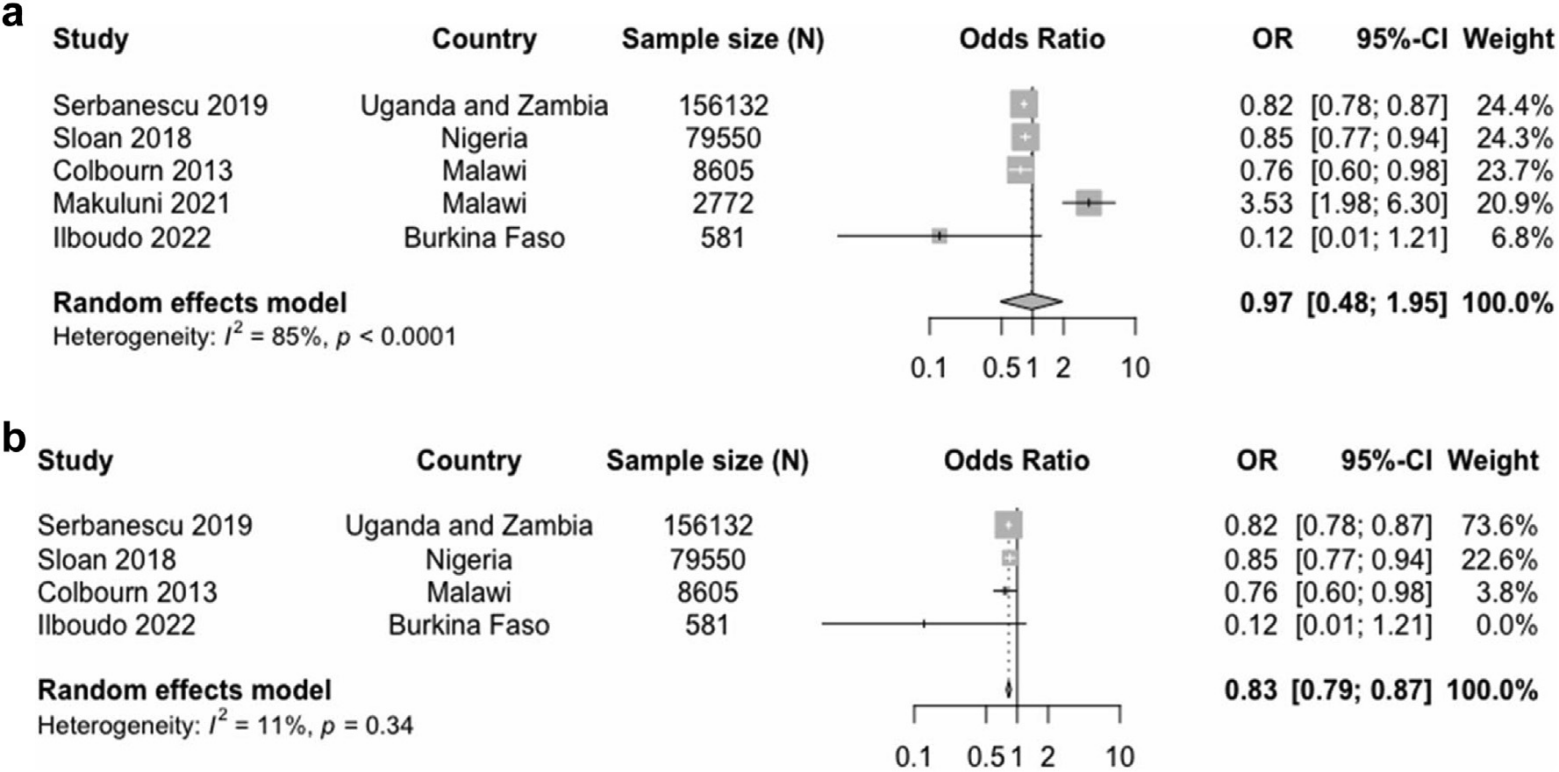
Forest plot of odds of stillbirths in intervention arm compared with control hospitals and communities in (a) all studies (b) without high risk of bias study.

The effect of studies that had both community and health facility interventions for stillbirth prevention was explored further. Five of such studies were identified and included in this analysis. Two studies each had a fair and good quality rating, while one study had a poor rating [Table 3]. The Colbourn study^22^ had four arms, one of which was a community and health facility intervention; the effect estimate from this arm was included in this analysis. The pooled estimate from these studies showed no evidence of a reduction in the odds of stillbirth (OR 0.97; 95% CI 0.48–1.95, n = 247,640) (Fig. 6). A sensitivity analysis excluding the Makuluni study,^37^ which had high risk of bias, showed significant evidence of reduced odds of stillbirths in the intervention group compared with the control group (OR 0.83; 95% CI 0.79–0.87, n = 244,868).

**Table 3:** Characteristics of studies with community and health facility interventions included in the meta-analysis.

	Authors and year	Country	Study design	Type of intervention	Intervention strategy	Quality rating
1	Serbanescu, 2019	Uganda and Zambia	Pre-post	• Knowledge and behavioural • Increasing access to skilled childbirth	• Women groups/peer counselling • Volunteer and community Health worker training • Community mobilisation	Fair
2	Sloan, 2018	Nigeria	Pre-post	• Knowledge and behavioural • Increasing access to skilled childbirth	• Women groups/peer counselling • Volunteer and community Health worker training • Community mobilisation	Fair
3	Colbourn, 2013	Malawi	cRCT	• Knowledge and behavioural • Increasing access to skilled childbirth	• Women groups/peer counselling • Volunteer and community Health worker training • Community mobilisation	Good
4	Makuluni, 2021	Malawi	Cross sectional	• Increasing access to skilled childbirth	• Transportation vouchers • Health worker training	Poor
5	Ilboudo, 2022	Burkina Faso	cRCT	• Nutritional	• Home visits • Volunteer and community Health worker training	Good

cRCT: cluster randomised controlled trial; nrCT: non-randomised controlled trial; RCT: randomised controlled trial.

## Discussion

This systematic review and meta-analysis evaluated the effectiveness of community-based interventions on reducing stillbirths in SSA. A total of 17 studies, conducted in 15 countries in SSA were included. The quality of included studies ranged from poor to good, with majority receiving a fair rating. While community-based interventions did not have a significant effect on reducing stillbirth, combined community and hospital-based interventions were found to significantly reduce the odds of stillbirth in intervention communities by 17%. A range of intervention types and delivery strategies were evaluated and there was no clear indication that a particular type of intervention or delivery strategy was more effective than others.

Our search identified 39 reports on community-based interventions which reported stillbirths as a primary or secondary outcome, however, only 17 of these reports quantitatively analysed the effectiveness of interventions for reducing stillbirths. Three previous reviews have assessed the effect of community-based interventions on maternal and newborn health with only one focusing solely on stillbirths.^7^^,^^14^^,^^15^ Evidence synthesis from studies across the world suggest different level of effectiveness of the interventions implemented for stillbirth prevention at the various stages of pregnancy.^7^^,^^40^, ^41^, ^42^ All three previous reviews curated several community-based interventions for stillbirth prevention globally, with one focusing solely in Africa.^15^ Two of the reviews assessed intervention effect on stillbirth and some show promising results (community financing schemes, maternal waiting homes),^7^ while some demonstrated significant reductions of stillbirth (training for newborn resuscitation, perinatal death audits, community mobilisation, health education).^7^^,^^14^ Our review showed that community-based interventions for preventing stillbirths were adopted in many countries across SSA, potentially due to increasing policy attention for stillbirth in this region.^43^^,^^44^

Effectiveness of community interventions depend on several factors including community readiness to adopt interventions, acceptability of interventions by women, robustness of implementation, strength of local administrative systems, local health system support, available infrastructure, quality of relationship between implementers and stakeholders, as well as sociocultural norms in the society which could preclude participation in the intervention.^45^ In assessing pooled statistical effect of these interventions, studies with different levels of implementation variability and methodological flaws are often pooled together and this remains a much-debated practice in research, especially with the inclusion of observational studies.^46^ Another common challenge in meta-analysis of effects of different types of intervention is the poor reporting in published literature, and the uncertainty about study characteristics omitted by some authors which limit effective moderator analysis.^47^ The summary effect of community-based intervention from this review showed a 4% reduction in the odds of stillbirth, although only five papers had good methodological quality while the other eight had moderate to serious methodological flaws. Some of the studies in the review suggest that he lack of effect on stillbirth may be due to factors like implementation effect (increased reporting of stillbirth due to intervention project),^25^ other ongoing national or regional projects in the control region or other factors which researchers are unable to explain.^28^^,^^48^ In a much larger Cochrane review of community-based interventions, there was a significant 19% reduction in stillbirth in intervention communities,^14^ however, some of the included papers had some health facility components included in the interventions. Conforming to the Cochrane study, meta-analysis of the joint community and health-facility interventions included in this review, were found to significantly lower the odds of stillbirth by 17%, whereas community-based intervention alone did not have a significant effect.

In SSA, several risk factors, which could be addressed by altering nutrition and behaviour of women, have been associated with increased risk of stillbirths including anaemia in pregnancy, malaria, gestational diabetes, hypertensive disorders of pregnancy, and external trauma.^49^, ^50^, ^51^ Knowledge and behavioural interventions included in this meta-analysis led to an insignificant 13% reduction in stillbirth, while nutritional interventions had no changes. These findings appear not to be unique to our study as similar results have been reported elsewhere.^52^ In a systematic review of global nutritional and behavioural interventions targeting stillbirths, no clear benefit of these interventions were found in reducing stillbirth, although many of the interventions led to increased demand for antenatal care services.^52^ Again this lack of effect may be due to the short implementation and follow-up period of such intervention programmes, under powered studies, methodical flaws or due to the inadequate integration of such programmes into the healthcare system.^53^ Another possible reason for a marginal improvement in stillbirths for community-based interventions could be the high proportion of stillbirths that occur during labour—an estimated 40% of all stillbirths globally and 50% in SSA.^2^

Intervention strategies aimed at improving access to skilled birth attendance had varying degrees of success including emergency transport, health worker training, traditional birth attendant (TBA) training, free maternal health services, community midwifery among others. Due to the continued patronage of TBAs by women, many interventions in some countries have focused on upskilling TBAs through periodic training to expand and improve their capacity to deliver basic primary obstetric care. The benefits of TBA training has been contested in the literature,^31^^,^^54^^,^^55^ while our review showed a non-significant 14% reduction in odds of stillbirth.

Interventions targeting the prevention of maternal infections associated with stillbirths (e.g., malaria, HIV, syphilis) were identified in this review. While the infection prevention studies in this meta-analysis showed no significant effect, another meta-analysis showed syphilis and malaria treatment in pregnancy have been beneficial in reducing stillbirth, decreasing oddss of stillbirths by 80% and 20%, respectively.^56^ This difference in finding may be due to the smaller number of studies (n = 2) and participants included in our review, which focused on SSA alone, whereas Ishaque et al.’s^56^ review had a larger sample size and included 25 studies from different parts of the world.^56^ Similarly, interventions delivered via mHealth, volunteer and community worker training, community midwifery and home visits led to non-significant reductions in the odds of stillbirth in the intervention communities, possibly due to the number of participants and the number of studies included. On the other hand, women’s group interventions included in this review did not lead to any changes in the odds of stillbirth. This finding is not unique to our review as similar results were reported in India^57^ and also by Prost and colleagues in a meta-analysis of effects of women’s groups on maternal and newborn health outcomes.^58^ It was interesting to note that while both interventions reported no effect on stillbirths, women’s group interventions positively affected maternal deaths and newborn deaths,^57^^,^^58^ raising the question about what changes need to be effected in women’s groups to extend the benefits to stillbirths.

An important finding from this review is that implementing community-based interventions alone without strengthening the quality and capacity of health facilities might not have a substantial effect on reducing stillbirths in SSA. In another review, Lassi and colleagues concluded that unless the primary care system and community actors effectively work together, neither of them will be able to maximise or leverage benefits the other has, thus reducing maximum potential impact.^59^ The achievement of the Millennium development goal 4 and 5 in Pakistan were also largely attributed to the successful merger between the community-based efforts and the formal health system.^59^ Therefore, as countries in this region plan and implement policies for reducing stillbirths, evidence provided by this study suggest that integrated efforts rather than community-only intervention are more effective.

This review needs to be interpreted bearing the following limitations in mind. First, only recent interventions documented and published in peer-reviewed journals and the grey literature from 2000 to July 7, 2023 were included in this review. It is possible that there were successful community interventions conducted prior to 2000 or documented in paper records that are inaccessible and therefore not included in this review. The studies that were included were categorized into community-based interventions or those that had a health facility component. This classification was based on the information gathered from the retrieved articles. However, there is a possibility that some studies were misclassified due to incomplete information reporting in the articles. Also, some interventions may have implemented changes in health facilities, but were not reported in published articles.

Moreover, some retrieved studies assessed the effect of interventions on the composite indicator perinatal death, which included stillbirths and early neonatal deaths, however, these studies were excluded from the analysis. Arguably, differentiating intervention effect on stillbirth from perinatal death is important for several reasons including the difference in timing of both events (stillbirths—28 weeks to time of labour, early neonatal death—first seven days of life) and therefore, the decision to exclude studies that had composite effect only is also a strength of this review. This is because, time-specific risk factors and their prevention strategies can only apply to one and not the other outcome when it comes to reducing perinatal death. For instance, implementing strategies during pregnancy to prevent maternal infections and stillbirths will not necessarily address the issue of early neonatal deaths caused by inadequate neonatal resuscitation. Instead, providing access to high-quality neonatal care can help prevent these early neonatal deaths. Also, separating stillbirths from perinatal deaths helps in accurately measuring and evaluating the impact of interventions.

Lastly, the limited number of studies included in the meta-analysis and the high degree of heterogeneity in the subgroup analysis limits the possible conclusions from this review. The quality of studies included in the analysis could also be a limitation especially for the subgroup analysis. While this does not mean that the included studies do not add useful information to the critical narrative synthesis, the synthesised evidence would have been more robust if all the included studies had been methodologically rigorous.

Overall, the review signalled the importance of integrated efforts in reducing stillbirths and may serve as a basis for formulating evidence-based policies and guidelines for stillbirth prevention.

In conclusion, this review suggests that harnessing the combined benefit of simultaneous interventions within health facilities and their surrounding communities could lead to significant reductions in stillbirths if properly planned, implemented, and scaled within the health system. More robust evaluations of community-based interventions and controlled trials are needed to understand the effect of interventions for reducing stillbirths. Also, many studies report stillbirth effects as composite perinatal mortality, future studies may benefit from a disaggregation of stillbirths from early neonatal deaths, to identify what specific interventions are beneficial for stillbirth reduction.

## Contributors

UGA, MN, CO, and JJK conceptualised this review. NR was responsible for library resources and supervision of the resource retrieval from databases. NR and UGA conducted the relevant searches. UGA, YYB, and AO were responsible for data extraction, verification, and analysis. CO, JJK, MN, and NR were responsible for validation and supervision. UGA prepared the initial draft of the paper, and all authors contributed to the development and refinement of subsequent drafts. UGA, YYB, AO, MN, CO, NR, and JJK read and approved the final manuscript for submission.

## Data sharing statement

All the data relevant to this study have been included in the article or included as Supplementary Information.

## Declaration of interests

As part of UGA's DPhil research funding, the Nuffield Department of Population Health, Balliol College and the Clarendon Fund cover her cost for attending meetings and conferences. MN also receives meetings and conference cost support from the University of Oxford and the Medical Research council. All other authors declare no competing interests.

## Appendix A Supplementary data

Supplementary Material

Data Extraction Sheet and Data From Included Studies
