# Supplementary material for: Effects of community-based interventions for stillbirths in sub-Saharan Africa: a systematic review and meta-analysis
Source: eClinicalMedicine. 2023 Dec 22;67:102386. doi: 10.1016/j.eclinm.2023.102386 (PMC10751841; doi:10.1016/j.eclinm.2023.102386)
Supplement: Supplementary Material [file mmc1.pdf]

Supplementary Material

Table of Contents

Appendix 1: Detailed search strategy 2

Appendix 2: Quality assessment of included studies.. 9

Appendix 1: Detailed search strategy

CINAHL(EBSCOhost)[1982-present]	
#	Query
S19	S4 AND S12 AND S15 AND S18
S18	S16 OR S17
S17	TX Angola or Benin or Botswana or "Burkina Faso" or Burundi or Cameroon or "Cape Verde" or "Cabo verde" or "Central African Republic" or Chad or Comoros or Congo or Djibouti or "Equatorial Guinea" or Eritrea or Ethiopia or Gabon or Gambia or Ghana or Guinea or "Ivory Coast" or "Cote d'Ivoire" or Jamahiriya or Jamahiryia or Kenya or Lesotho or Liberia or Libya or Libia or Madagascar or Malawi or Mali or Mauritania or Mauritius or Mayote or Mozambique or Mocambique or Namibia or Niger or Nigeria or Principe or Reunion or Rwanda or "Sao Tome" or Senegal or Seychelles or "Sierra Leone" or Somalia or "South Africa" or "St Helena" or Sudan or Swaziland or Tanzania or Togo or Tunisia or Uganda or "Western Sahara" or Zaire or Zambia or Zimbabwe or "Central Africa" or "Central African" or "West Africa" or "West African" or "Western Africa" or "Western African" or "East Africa" or "East African" or "Eastern Africa" or "Eastern African" or "South African" or "Southern Africa" or "Southern African" or "sub Saharan Africa" or "sub Saharan African" or "subSaharan Africa" or "subSaharan African"
S16	(MH "Africa") OR (MH "Africa South of the Sahara+")
S15	S13 OR S14
S14	TI ((stillbirth* or ((perinatal or fetal or foetal or fetus or intrauterine or intra-uterine) N1 (death* or mortality)))) OR AB ((stillbirth* or ((perinatal or fetal or foetal or fetus or intrauterine or intra-uterine) N1 (death* or mortality))))
S13	(MH "Perinatal Death") OR (MH "Pregnancy Complications/MO") OR (MH "Pregnancy Outcomes")
S12	S5 OR S6 OR S7 OR S8 OR S9 OR S10 OR S11
S11	TI (((community N2 intervention*) or community mobilization or community mobilisation or birth attendant* or community health worker* or community worker* or home visit* or women* groups or mother* groups or mobile health or mhealth or m-health or mobile phone* or cellphone* or cell phone* or smartphone* or health promotion or health education or national program* or nutritional intervention* or nutritional program* or smoking cessation or ((stop* or quit*) N2 smok*))) OR AB (((community N2 intervention*) or community mobilization or community mobilisation or birth attendant* or community health worker* or community worker* or home visit* or women* groups or mother* groups or mobile health or mhealth or m-health or mobile phone* or cellphone* or cell phone* or smartphone* or health promotion or health education or national program* or nutritional intervention* or nutritional program* or smoking cessation or ((stop* or quit*) N2 smok*)))
S10	(MH "Cellular Phone+")
S9	(MH "Smoking Cessation") OR (MH "Smoking Cessation Programs")
S8	(MH "National Health Programs")
S7	(MH "Health Education") OR (MH "Nutrition Education") OR (MH "Health Promotion")
S6	(MH "Midwives+") OR (MH "Community Health Workers")
S5	(MH "Community Health Services") OR (MH "Community Programs") OR (MH "Community Health Centers")
S4	S1 OR S2 OR S3
S3	TI (pregnan* or maternal care) OR AB (pregnan* or maternal care)

S2	(MH "Maternal Health Services+")
S1	(MH "Pregnancy") OR (MH "Expectant Mothers")

**Web of Science Core Collection – Science Citation Index and Social Science Citation index [1900-present]**	
#	Query
1	TS=(pregnan* or maternal care)
2	TS=((community NEAR/2 intervention*) or community mobilization or community mobilisation or birth attendant* or community health worker* or community worker* or home visit* or women* groups or mother* groups or mobile health or mhealth or m-health or mobile phone* or cellphone* or cell phone* or smartphone* or health promotion or health education or national program* or nutritional intervention* or nutritional program* or smoking cessation or ((stop* or quit*) NEAR/2 smok*))
3	TS=(stillbirth* or ((perinatal or fetal or foetal or fetus or intrauterine or intra-uterine) NEXT (death* or mortality)))
4	TS=(Angola or Benin or Botswana or "Burkina Faso" or Burundi or Cameroon or "Cape Verde" or "Cabo verde" or "Central African Republic" or Chad or Comoros or Congo or Djibouti or "Equatorial Guinea" or Eritrea or Ethiopia or Gabon or Gambia or Ghana or Guinea or "Ivory Coast" or "Cote d'Ivoire" or Jamahiriya or Jamahiryia or Kenya or Lesotho or Liberia or Libya or Libia or Madagascar or Malawi or Mali or Mauritania or Mauritius or Mayote or Mozambique or Mocambique or Namibia or Niger or Nigeria or Principe or Reunion or Rwanda or "Sao Tome" or Senegal or Seychelles or "Sierra Leone" or Somalia or "South Africa" or "St Helena" or Sudan or Swaziland or Tanzania or Togo or Tunisia or Uganda or "Western Sahara" or Zaire or Zambia or Zimbabwe or "Central Africa" or "Central African" or "West Africa" or "West African" or "Western Africa" or "Western African" or "East Africa" or "East African" or "Eastern Africa" or "Eastern African" or "South African" or "Southern Africa" or "Southern African" or "sub Saharan Africa" or "sub Saharan African" or "subSaharan Africa" or "subSaharan African") OR CU=(Angola or Benin or Botswana or "Burkina Faso" or Burundi or Cameroon or "Cape Verde" or "Cabo verde" or "Central African Republic" or Chad or Comoros or Congo or Djibouti or "Equatorial Guinea" or Eritrea or Ethiopia or Gabon or Gambia or Ghana or Guinea or "Ivory Coast" or "Cote d'Ivoire" or Jamahiriya or Jamahiryia or Kenya or Lesotho or Liberia or Libya or Libia or Madagascar or Malawi or Mali or Mauritania or Mauritius or Mayote or Mozambique or Mocambique or Namibia or Niger or Nigeria or Principe or Reunion or Rwanda or "Sao Tome" or Senegal or Seychelles or "Sierra Leone" or Somalia or "South Africa" or "St Helena" or Sudan or Swaziland or Tanzania or Togo or Tunisia or Uganda or "Western Sahara" or Zaire or Zambia or Zimbabwe or "Central Africa" or "Central African" or "West Africa" or "West African" or "Western Africa" or "Western African" or "East Africa" or "East African" or "Eastern Africa" or "Eastern African" or "South African" or "Southern Africa" or "Southern African" or "sub Saharan Africa" or "sub Saharan African" or "subSaharan Africa" or "subSaharan African")
5	#1 AND #2 AND #3 AND #4

**Medline (Ovid MEDLINE® Epub Ahead of Print, In-Process & Other Non-Indexed Citations, Ovid MEDLINE® Daily and Ovid MEDLINE®) 1946 to present**	
#	Query
1	Pregnancy/ or Pregnant Women/
2	exp Maternal Health Services/
3	(pregnan* or maternal care).mp.
4	1 or 2 or 3
5	community health services/ or community participation/
6	Midwifery/ or Community Health Workers
7	health education/ or health promotion/
8	National Health Programs/

9	Smoking Cessation/
10	exp Cell Phone/
11	((community adj2 (intervention* or program*)) or community mobilization or community mobilisation or midwives or midwifery or birth attendant* or community health worker* or community worker* or home visit* or women* groups or mother* groups or mobile health or mhealth or m-health or mobile phone* or cellphone* or cell phone* or smartphone* or text messag* or health promotion or health education or national program* or nutrition* intervention* or nutrition* program* or smoking cessation or ((stop* or quit*) adj2 smok*)).mp.
12	5 or 6 or 7 or 8 or 9 or 10 or 11
13	perinatal death/ or fetal death/ or stillbirth/
14	fetal mortality/ or perinatal mortality/
15	Pregnancy Outcome/
16	Pregnancy Complications/mo
17	(stillbirth* or ((perinatal or fetal or foetal or fetus or intrauterine or intra-uterine) adj (death? or mortality))).mp.
18	13 or 14 or 15 or 16 or 17
19	Africa/ or exp "Africa South of the Sahara"/
20	(Angola or Benin or Botswana or "Burkina Faso" or Burundi or Cameroon or "Cape Verde" or "Cabo verde" or "Central African Republic" or Chad or Comoros or Congo or Djibouti or "Equatorial Guinea" or Eritrea or Ethiopia or Gabon or Gambia or Ghana or Guinea or "Ivory Coast" or "Cote d'Ivoire" or Jamahiriya or Jamahiryia or Kenya or Lesotho or Liberia or Libya or Libia or Madagascar or Malawi or Mali or Mauritania or Mauritius or Mayote or Mozambique or Mocambique or Namibia or Niger or Nigeria or Principe or Reunion or Rwanda or "Sao Tome" or Senegal or Seychelles or "Sierra Leone" or Somalia or "South Africa" or "St Helena" or Sudan or Swaziland or Tanzania or Togo or Tunisia or Uganda or "Western Sahara" or Zaire or Zambia or Zimbabwe or "Central Africa" or "Central African" or "West Africa" or "West African" or "Western Africa" or "Western African" or "East Africa" or "East African" or "Eastern Africa" or "Eastern African" or "South African" or "Southern Africa" or "Southern African" or "sub Saharan Africa" or "sub Saharan African" or "subSaharan Africa" or "subSaharan African").mp.
21	19 or 20
22	4 and 12 and 18 and 21

WHO Global Index Medicus – limit to AIM (**globalindexmedicus.net)**	
#	Query
1	(pregnan* OR maternal) AND (community OR "birth attendant*" OR "home visit*" OR "womens groups" OR "mothers groups" OR "mobile health" OR mhealth OR m-health OR "mobile phone*" OR cellphone* OR "cell phone*" OR smartphone* OR "health promotion" OR "health education" OR "national program*" OR "nutritional intervention*" OR "nutrition intervention*" OR "nutrition program*" OR "nutritional program*"<br>OR "smoking cessation" OR "stop smoking" OR "quit smoking") AND (stillbirth* OR "perinatal death*" OR "perinatal mortality" OR "fetal death*" OR "fetal mortality" OR "foetal death*" OR "foetal mortality" OR "fetus death*" OR "fetus mortality" OR "foetus death*" OR "foetus mortality" OR "intrauterine death*" OR "intrauterine mortality" OR "intra-uterine death*" OR "intra-uterine mortality")

**Global Health 1973 to present**	
#	Query
1	Pregnancy/ or Pregnant Women/
2	(pregnan* or maternal care).mp.
3	1 or 2

4	community health/ or community health services/ or community involvement/ or community nutrition/ or community programmes/
5	community health workers/ or midwives/ or traditional birth attendants/
6	health education/ or health promotion/
7	health programmes/
8	Smoking Cessation/
9	mobile telephones/
10	nutrition programmes/ or nutritional interventions/
11	((community adj2 (intervention* or program*)) or community mobilization or community mobilisation or midwives or midwifery or birth attendant* or community health worker* or community worker* or home visit* or women* groups or mother* groups or mobile health or mhealth or m-health or mobile phone* or cellphone* or cell phone* or smartphone* or text messag* or health promotion or health education or national program* or nutrition* intervention* or nutrition* program* or smoking cessation or ((stop* or quit*) adj2 smok*)).mp.
12	4 or 5 or 6 or 7 or 8 or 9 or 11
13	fetal death/ or stillbirths/
14	perinatal mortality/
15	(stillbirth* or ((perinatal or fetal or foetal or fetus or intrauterine or intra-uterine) adj (death? or mortality))).mp.
16	13 or 14 or 15
17	exp "africa south of sahara"/
18	(Angola or Benin or Botswana or "Burkina Faso" or Burundi or Cameroon or "Cape Verde" or "Cabo verde" or "Central African Republic" or Chad or Comoros or Congo or Djibouti or "Equatorial Guinea" or Eritrea or Ethiopia or Gabon or Gambia or Ghana or Guinea or "Ivory Coast" or "Cote d'Ivoire" or Jamahiriya or Jamahiryia or Kenya or Lesotho or Liberia or Libya or Libia or Madagascar or Malawi or Mali or Mauritania or Mauritius or Mayote or Mozambique or Mocambique or Namibia or Niger or Nigeria or Principe or Reunion or Rwanda or "Sao Tome" or Senegal or Seychelles or "Sierra Leone" or Somalia or "South Africa" or "St Helena" or Sudan or Swaziland or Tanzania or Togo or Tunisia or Uganda or "Western Sahara" or Zaire or Zambia or Zimbabwe or "Central Africa" or "Central African" or "West Africa" or "West African" or "Western Africa" or "Western African" or "East Africa" or "East African" or "Eastern Africa" or "Eastern African" or "South African" or "Southern Africa" or "Southern African" or "sub Saharan Africa" or "sub Saharan African" or "subSaharan Africa" or "subSaharan African").mp.
19	Africa/ or exp "Africa South of the Sahara"/
20	17 or 18
21	3 and 12 and 16 and 19

**Embase 1974 to present**	
#	Query
1	Pregnancy/ or Pregnant Woman/
2	Maternal Health Service/
3	(pregnan* or maternal care).mp.
4	1 or 2 or 3
5	community care/ or community program/ or community participation/
6	midwife/ or traditional birth attendant/ or health auxiliary/
7	health education/ or health promotion/
8	health program/
9	Smoking Cessation/
10	exp mobile phone/
11	((community adj2 intervention*) or community mobilization or community mobilisation or birth attendant* or community health worker* or community worker* or home visit* or women* groups

	or mother* groups or mobile health or mhealth or m-health or mobile phone* or cellphone* or cell phone* or smartphone* or health promotion or health education or national program* or nutritional intervention* or nutritional program* or smoking cessation or ((stop* or quit*) adj2 smok*)).mp.
12	5 or 6 or 7 or 8 or 9 or 10 or 11
13	exp fetus death/ or perinatal death/
14	fetus mortality/ or exp perinatal mortality/ or prenatal mortality/
15	*Pregnancy Outcome/
16	(stillbirth* or ((perinatal or fetal or foetal or fetus or intrauterine or intra-uterine) adj (death? or mortality))).mp.
17	13 or 14 or 15 or 16
18	exp "africa south of the sahara"/ or africa/
19	(Angola or Benin or Botswana or "Burkina Faso" or Burundi or Cameroon or "Cape Verde" or "Cabo verde" or "Central African Republic" or Chad or Comoros or Congo or Djibouti or "Equatorial Guinea" or Eritrea or Ethiopia or Gabon or Gambia or Ghana or Guinea or "Ivory Coast" or "Cote d'Ivoire" or Jamahiriya or Jamahiryia or Kenya or Lesotho or Liberia or Libya or Libia or Madagascar or Malawi or Mali or Mauritania or Mauritius or Mayote or Mozambique or Mocambique or Namibia or Niger or Nigeria or Principe or Reunion or Rwanda or "Sao Tome" or Senegal or Seychelles or "Sierra Leone" or Somalia or "South Africa" or "St Helena" or Sudan or Swaziland or Tanzania or Togo or Tunisia or Uganda or "Western Sahara" or Zaire or Zambia or Zimbabwe or "Central Africa" or "Central African" or "West Africa" or "West African" or "Western Africa" or "Western African" or "East Africa" or "East African" or "Eastern Africa" or "Eastern African" or "South African" or "Southern Africa" or "Southern African" or "sub Saharan Africa" or "sub Saharan African" or "subSaharan Africa" or "subSaharan African").mp.
20	18 or 19
21	4 and 12 and 17 and 20

**ProQuest Dissertations & Theses Global (proquest.com)**	
#	Query
1	noft((pregnan* or "maternal care")) AND noft(((community NEAR2 intervention*) or community mobilization or community mobilisation or birth attendant* or community health worker* or community worker* or home visit* or women* groups or mother* groups or mobile health or mhealth or m-health or mobile phone* or cellphone* or cell phone* or smartphone* or health promotion or health education or national program* or nutritional intervention* or nutritional program* or smoking cessation or ((stop* or quit*) NEAR2 smok*))) AND noft((stillbirth* or ((perinatal or fetal or foetal or fetus or intrauterine or intra-uterine) NEAR2 (death* or mortality))))

**Cochrane Central Register of Controlled Trials(Cochrane Library, Wiley)[1ssue 7 of 12, July 2022]**	
#	Query
1	MeSH descriptor: [Pregnancy] this term only
2	MeSH descriptor: [Pregnant Women] explode all trees
3	MeSH descriptor: [Maternal Health Services] explode all trees
4	#1 OR #2 OR #3
5	MeSH descriptor: [Community Health Services] this term only
6	MeSH descriptor: [Community Participation] this term only
7	MeSH descriptor: [Community Health Workers] explode all trees
8	MeSH descriptor: [Midwifery] explode all trees
9	MeSH descriptor: [Health Promotion] this term only
10	MeSH descriptor: [Health Education] this term only
11	MeSH descriptor: [National Health Programs] this term only
12	MeSH descriptor: [Smoking Cessation] explode all trees
13	MeSH descriptor: [Cell Phone] explode all trees

14	(((community NEAR/2 intervention*) or community mobilization or community mobilisation or birth attendant* or community health worker* or community worker* or home visit* or women* groups or mother* groups or mobile health or mhealth or m-health or mobile phone* or cellphone* or cell phone* or smartphone* or health promotion or health education or national program* or nutritional intervention* or nutritional program* or smoking cessation or ((stop* or quit*) NEAR/2 smok*))):ti,ab,kw
15	#5 OR #6 OR #7 OR #8 OR #9 OR #10 OR #11 OR #12 OR #13 OR #14
16	MeSH descriptor: [Perinatal Death] explode all trees
17	MeSH descriptor: [Fetal Death] explode all trees
18	MeSH descriptor: [Perinatal Mortality] explode all trees
19	MeSH descriptor: [Fetal Mortality] explode all trees
20	((stillbirth* or ((perinatal or fetal or foetal or fetus or intrauterine or intra-uterine) NEXT (death* or mortality)))):ti,ab,kw
21	#16 OR #17 OR #18 OR #19 OR #20
22	#4 AND #15 AND #21
23	MeSH descriptor: [Africa South of the Sahara] explode all trees
24	MeSH descriptor: [Africa] this term only
25	(Angola or Benin or Botswana or "Burkina Faso" or Burundi or Cameroon or "Cape Verde" or "Cabo verde" or "Central African Republic" or Chad or Comoros or Congo or Djibouti or "Equatorial Guinea" or Eritrea or Ethiopia or Gabon or Gambia or Ghana or Guinea or "Ivory Coast" or "Cote d'Ivoire" or Jamahiriya or Jamahiryia or Kenya or Lesotho or Liberia or Libya or Libia or Madagascar or Malawi or Mali or Mauritania or Mauritius or Mayote or Mozambique or Mocambique or Namibia or Niger or Nigeria or Principe or Reunion or Rwanda or "Sao Tome" or Senegal or Seychelles or "Sierra Leone" or Somalia or "South Africa" or "St Helena" or Sudan or Swaziland or Tanzania or Togo or Tunisia or Uganda or "Western Sahara" or Zaire or Zambia or Zimbabwe or "Central Africa" or "Central African" or "West Africa" or "West African" or "Western Africa" or "Western African" or "East Africa" or "East African" or "Eastern Africa" or "Eastern African" or "South African" or "Southern Africa" or "Southern African" or "sub Saharan Africa" or "sub Saharan African" or "subSaharan Africa" or "subSaharan African"):ti,ab,kw
26	#24 OR #25
27	#22 AND #26

**WHO International Clinical Trials Registry** https://trialsearch.who.int/	
#	Query
1	Pregnancy OR Pregnant (title row) AND Community(intervention row)
2	(pregnant OR pregnancy) AND community AND (stillbirth OR stillbirths OR fetal death OR fetal deaths OR fetal mortality OR perinatal death OR perinatal deaths OR perinatal mortality)

**ClinicalTrials** https://www.clinicaltrials.gov/	
#	Query
1	Stillbirth OR Perinatal death OR Perinatal mortality OR fetal death OR fetal mortality(title row) AND Community(intervention row)
2	Pregnancy OR Pregnant(Condition row) AND Community (intervention row)
3	(pregnant OR pregnancy) AND community AND (stillbirth OR stillbirths OR fetal death OR fetal deaths OR fetal mortality OR perinatal death OR perinatal deaths OR perinatal mortality) (Other terms)

**Google**	
#	Query
1	(pregnancy OR pregnant) AND (community OR "birth attendants" OR "home visits" OR "womens groups" OR "mothers groups" OR "mobile health" OR mhealth OR m-health OR "mobile phones" OR cellphones OR "cell phones" OR smartphones OR "health promotion" OR "health education" OR "nutritional interventions" OR "nutrition interventions" OR programs OR programmes OR "smoking cessation" OR "stop smoking" OR "quit smoking") AND (stillbirths OR "perinatal deaths" OR "perinatal mortality" OR "fetal deaths" OR "fetal mortality" OR "foetal deaths" OR "foetal mortality" OR "fetus deaths" OR "fetus mortality" OR "foetus deaths" OR "foetus mortality" OR "intrauterine deaths" OR "intrauterine mortality" OR "intra-uterine deaths" OR "intrauterine mortality")
2	(pregnancy OR pregnant) AND (community OR "birth attendants" OR "home visits" OR mhealth OR "mobile phones" OR "health education" OR "nutritional interventions" OR programs OR "smoking cessation" AND (stillbirths OR "perinatal deaths") site:.org

Appendix 2: Quality assessment of included studies

Quality assessment for Pre-post studies - National Heart, Lung, and Blood Institute Appraisal Checklist

S/N	First author and year	1	2	3	4	5	6	7	8	9	11	12	Total
1	Sloan 2018	1	1	1	1	1	0	1	0	1	1	1	8(75)
2	Serbanescu 2019	1	1	1	0	1	0	1	0	0	0	0	5(45)
3	Shikuku 2020	1	0	0	0	0	1	1	0	1	0	0	5(45)

1. Was the research question or objective in this paper clearly stated?
2. Were eligibility/selection criteria for the study population prespecified and clearly described?
3. Were the participants in the study representative of those who would be eligible for the test/service/intervention in the general or clinical population of interest?
4. Were all eligible participants that met the prespecified entry criteria enrolled?
5. Was the sample size sufficiently large to provide confidence in the findings?
6. Was the test/service/intervention clearly described and delivered consistently across the study population?
7. Were the outcome measures prespecified, clearly defined, valid, reliable, and assessed consistently across all study participants?
8. Were the people assessing the outcomes blinded to the participants' exposures/interventions?
9. Did the statistical methods examine changes in outcome measures from before to after the intervention? Were statistical tests done that provided p values for the pre-to-post changes?
11. Were outcome measures of interest taken multiple times before the intervention and multiple times after the intervention (i.e., did they use an interrupted time-series design)?
12. If the intervention was conducted at a group level (e.g., a whole hospital, a community, etc.) did the statistical analysis take into account the use of individual-level data to determine effects at the group level?

1: fully reported; 0: Not reported; N/A: Not applicable

Quality assessment for cross-sectional and cohort studies - National Heart, Lung, and Blood Institute

S/N	First author and year	1	2	3	4	5	6	7	8	9	10	11	12	13	14	Total (%)
1	Fatti 2016	1	1	0	1	0	1	1	0	1	0	1	0	0	1	8(57)
2	Makuluni 2021	1	1	0	1	0	1	1	0	0	0	1	0	0	0	6(43)

1. Was the research question or objective in this paper clearly stated?
2. Was the study population clearly specified and defined?
3. Was the participation rate of eligible persons at least 50%?
4. Were all the subjects selected or recruited from the same or similar populations (including the same time period)? Were inclusion and exclusion criteria for being in the study prespecified and applied uniformly to all participants?
5. Was a sample size justification, power description, or variance and effect estimates provided?
6. For the analyses in this paper, were the exposure(s) of interest measured prior to the outcome(s) being measured?
7. Was the timeframe sufficient so that one could reasonably expect to see an association between exposure and outcome if it existed?

	8. For exposures that can vary in amount or level, did the study examine different levels of the exposure as related to the outcome (e.g., categories of exposure, or exposure measured as continuous variable)?<br>9. Were the exposure measures (independent variables) clearly defined, valid, reliable, and implemented consistently across all study participants?<br>10. Was the exposure(s) assessed more than once over time?<br>11. Were the outcome measures (dependent variables) clearly defined, valid, reliable, and implemented consistently across all study participants?<br>12. Were the outcome assessors blinded to the exposure status of participants?<br>13. Was loss to follow-up after baseline 20% or less?<br>14. Were key potential confounding variables measured and adjusted statistically for their impact on the relationship between exposure(s) and outcome(s)?1: fully reported; 0: Not reported; N/A: Not applicable

Quality assessment for (cluster) Randomised control trials –Cochrane Risk of Bias II assessment tool

S/N	First author and year	1a	1b	2	3	4	5	6
1	Mohammed 2016	High	Some concerns	Low	Low	Low	Low	Some concerns
2	Colbourn 2013	Low	Low	Low	Low	Low	Low	Low
3	Lewycka 2013	Some concerns	Low	Low	Low	High	Low	Some concerns
4	Lund 2014	Some concerns	Low	Low	Low	Low	Low	Some concerns
5	Alexander 2018	Low	Low	Low	Low	Low	Low	Low
6	Matendo 2011	Some concerns	Low	Low	Low	Low	Low	Some concerns
7	Scott 2019	Low	Low	Low	Low	Low	Low	Low
8	Ilboudo et al 2022	Low	Some concerns	Low	Low	Low	Low	Some concerns
9	Kone et al 2022	Low	Low	Low	Low	Low	Low	Low
10	De Kok et al 2022	Low	Low	Low	Low	Low	Low	Low

Domain 1a. Randomization process
Domain 1b: Risk of bias arising from the timing of identification or recruitment of participants
Domain 2. Deviations from intended interventions
Domain 3. Missing outcome data
Domain 4. Measurement of the outcome
Domain 5. Selection of the reported result
Domain 6. Overall Bias
